# Withdrawal: Transformer-based comparative multi-view illegal transaction detection

**DOI:** 10.1371/journal.pone.0283785

**Published:** 2023-04-10

**Authors:** The PLOS ONE Editors

This article [1] was published in error by PLOS after the authors requested withdrawal. Therefore, *PLOS ONE* has withdrawn this article [1] and removed the article contents from the journal website. The publisher apologizes for the error.
